# Supplementary material for: A Quality Assurance Discrimination Tool for the Evaluation of Satellite Laboratory Practice Excellence in the Context of the European Official Meat Inspection for Trichinella spp
Source: Foods. 2023 Nov 20;12(22):4186. doi: 10.3390/foods12224186 (PMC10670484; doi:10.3390/foods12224186)
Supplement: Supplementary file 1 [file foods-12-04186-s001.zip › Table S3.pdf]

Table S3. Classification of deviations and finding examples obtained during audits.

Classification of findings affecting QMS requirements	
DEVIATIONS (According to severity of the finding)	Non-conformities (NC)<br>Observations (OB)
AMBIT (According to affected requirement)	*Technical Requirements (TR)*<br>*Management Requirements (MR)*
TYPES (Depending on affected component)	SUBTYPES
TYPE 1<br>Affects TECHNIQUE and/or trial information.	SUBTYPE (a) The teflon magnet is not the regulatory size (≈ 5 cm in length), it is 3 cm in length (OB-TR). The use of a second decanter (graduated funnel with safety key) is not considered regulatory (OB-TR). The sieve has a pore diameter of 177 microns, which does not comply with the EC Regulation 2075/2005 (NC-TR).<br>SUBTYPE (b) Not recording the percentage (%) of digestion (OB-TR). In the reports, the spaces reserved for each value are not used appropriately, writing values in spaces that do not correspond (OB-MR). Not recording the batch of the reagents (NC-TR). Not recording final results of the samples (NC-TR). Incorrect or lack of identification of samples (NC-TR).
TYPE 2<br>Affects EQUIPMENT, MATERIAL, REAGENTS	SUBTYPE (a) No calibration of equipment is carried out (OB-TR). There is no plan for the maintenance, verification and calibration of the equipment (MANCA Plan) (NC-TR). The record of the annual planning for maintenance, verification and calibration of the equipment is incomplete (OB-MR).<br>SUBTYPE (b) The heating plate display does not show tenths of a degree (OB-TR). The thermometer does not work correctly and is not identified as equipment out of use (OB-TR). The equipment technical sheet does not correspond to the equipment used during the procedure (NC-TR). The equipment is not correctly labeled, no calibration label on the equipment (OB-MR).<br>SUBTYPE (c) There is no graduated glass cylinder (50-100 ml) (OB-TR). The laboratory does not have thermometer (NC-TR).<br>SUBTYPE (d) The instruction of the heating plate is missing (OB-TR).<br>SUBTYPE (e) The graduated cylinder is broken or damaged, the container for collecting and inactivating liquid waste from positive samples is broken or damaged (OB-TR).<br>SUBTYPE (f) There is no concentrated chlorine-based reagent available to inactivate waste from positive samples (OB-TR). The reagent belongs to a different batch than that indicated in the record (OB-TR).
TYPE 3<br>Affects QUALIFICATION	NO SUBTYPES. The laboratory does not provide documents that ensure the proficiency level of technical staff performing the procedure (NC-TR).
TYPE 4<br>Affects QUALITY ASSURANCE	SUBTYPE (a) No internal or external quality controls are performed (OB-TR).<br>SUBTYPE (b) A non-conformity deviation identified during an internal quality control (i.e. > 5% of undigested tissue after a specific procedure) is not registered in the corresponding reports (OB-TR). Having identified deviations during internal audits, no corrective action plan is carried out (NC-TR)

	SUBTYPE (c) No calibration control of the weighing scale is performed (OB-TR), no calibration certificate of specific equipment is provided (NC-TR), calibration certificate is expired (NC-TR).<br><br>SUBTYPE (d) No providing the “Certificate of Analysis” of the hydrochloric acid (NC-TR).<br><br>SUBTYPE (e) The reagent supplier does not have the ISO 9001 certificate (OB-TR). It is unknown whether the supplier does or does not have the ISO 9001 certificate (OB-MR).<br><br>SUBTYPE (f). A corresponding document does not include the corresponding data (e.g., the document “list of deviations” regarding audit, does not include the deviations identified during the last audit) (OB-MR).<br><br>SUBTYPE (g) Pepsin is not correctly stored (temperature) (NC-TR).
TYPE 5<br><br>Affects RECORDS, FORMATS AND OTHER DOCUMENTS	SUBTYPE (a) The document of the list of suppliers is incomplete (OB-MR). There is no report of the reagents control (NC-MR).<br><br>SUBTYPE (b) Not using the correct format of the documentation to request reagents (NC-TR). There is no document for “reagents in use” (OB-MR).<br><br>SUBTYPE (c) Not filling in the reports correctly (e.g. negative samples indicated with a symbol (-) instead of the correct form (N)) (OB-TR).<br><br>SUBTYPE (d) The laboratory does not provide the current (in force) documents that include the applicable regulations (EU Regulation) of *Trichinella* sp. inspection (OB-TR).<br><br>SUBTYPE (e) The report showing the sample result shows deletions (OB-TR).<br><br>SUBTYPE (f) No keeping the receipts of the reagents (OB-MR).
TYPE 6<br><br>Affects OTHER components	SUBTYPE (a) The laboratory does not have drinking water (OB-TR) or cold water (NC-TR). The existing sink is not suitable for cleaning laboratory equipment, there is no correct ventilation system (OB-MR).<br><br>SUBTYPE (b) Using rusty materials, not using permanent marker to identify-label samples (OB-TR).<br><br>SUBTYPE (c) The container to collect the liquid waste from positive samples and used to wash-rinse the material does not have a capacity of between 10 and 15 liters (OB-TR).<br><br>SUBTYPE (d) Not proving the instructions of the equipment, the instructions are kept outside the laboratory (OB-MR). The instructions of the magnetic stirrer with heating plate is not in the native language of the technical operator, only the English version is available (NC-MR).
